# Intraoperative Radiotherapy as a Tumour-Bed Boost Combined with Whole Breast Irradiation Versus Conventional Radiotherapy in Patients with Early-Stage Breast Cancer: A Systematic Review and Meta-analysis

**DOI:** 10.1245/s10434-023-13955-w

**Published:** 2023-07-28

**Authors:** Jiafa He, Shengying Chen, Lingling Ye, Yang Sun, Yan Dai, Xue Song, Xiaojie Lin, Rui Xu

**Affiliations:** Breast Department, Guangdong Provincial Hospital of Chinese Medicine, The Second Affiliated Hospital of Guangzhou University of Chinese Medicine, Guangdong Provincial Academy of Chinese Medical Sciences, Guangzhou, Guangdong China

## Abstract

**Background:**

There is no definitive answer regarding the efficacy of intraoperative radiotherapy (IORT) as a tumour bed boost for patients with early-stage breast cancer. The purpose of this meta-analysis was to summarise the available evidence and explore the efficacy and safety of IORT combined with whole breast irradiation (WBI) versus conventional radiotherapy in women with early-stage breast cancer who underwent breast-conserving surgery.

**Methods:**

The PUBMED, MEDLINE, EMBASE, Web of Science, and Cochrane Library databases were searched from inception to December 31, 2022. We collected studies on the efficacy, cosmetic outcome, and safety of IORT boost combined with WBI compared with those of conventional radiotherapy in patients with early-stage breast cancer after breast-conserving surgery. Two authors independently performed the literature selection and data extraction. The quality of the randomised, controlled trials (RCTs) was assessed according to the PEDro scale. The quality of non-RCTs was assessed according to the Methodological Index for Non-Randomised Studies. Risk ratios (RRs) for the local recurrence rate (LRR), distant metastasis rate (DMR), disease-free survival (DFS), cosmetic outcome, and toxicity were pooled using fixed or random effects models. Meta-analysis of the included studies was performed by using RevMan 5.3 software.

**Results:**

Nine studies, including one RCT and eight non-RCTs, with a total of 3219 patients were included. In terms of LRR, there was no significant benefit of IORT boost+WBI over conventional radiotherapy (with or without the tumour bed boost) (RR = 0.77, 95% confidence interval (CI): 0.54–1.09, *P* = 0.14), but a trend towards benefit could be identified. There was a significant reduction in DMR in the IORT boost+WBI group (RR = 0.63, 95% CI: 0.46–0.85, *P* = 0.003) and a significant improvement in DFS (RR = 0.40, 95% CI: 0.25–0.65, *P* = 0.0002). Exploratory subgroup analysis showed that the DMR and DFS of the electron boost group were significantly better than those of conventional radiotherapy group, and there was a tendency for LRR to improve in the electron boost group. However, the LRR, DMR, and DFS did not effectively improve in the x-ray boost group. In terms of appearance and toxicity, there were no significant differences in cosmetic outcome, fibrosis, and hyperpigmentation between the two groups (RR = 0.99, 95% CI: 0.91–1.07, *P* = 0.78; RR = 1.02, 95% CI: 0.41–2.56, *P* = 0.96; RR = 0.42, 95% CI: 0.10–1.72, *P* = 0.23), but the incidence of oedema was significantly reduced in the IORT boost+WBI group (RR = 0.27, 95% CI: 0.13–0.59, *P* = 0.0009).

**Conclusions:**

IORT boost+WBI is more effective than conventional radiotherapy after breast-conserving surgery in patients with early-stage breast cancer, and electron boost exhibits better efficacy than x-ray boost. In addition, the cosmetic and safety profiles of IORT boost+WBI are not inferior to those of conventional radiotherapy.

**Supplementary Information:**

The online version contains supplementary material available at 10.1245/s10434-023-13955-w.

The 2020 global cancer data show that the incidence of breast cancer has surpassed that of lung cancer, making breast cancer the most common cancer worldwide.^1^ Breast-conserving surgery is now the internationally accepted treatment of choice for early-stage breast cancer. Studies have shown that the addition of radiation therapy after breast-conserving surgery for early-stage breast cancer can further improve local control rates and reduce the risk of local recurrence after surgery.^2,3^ Therefore, breast-conserving surgery combined with external beam radiotherapy (EBRT) is considered the “gold standard” treatment for early-stage breast cancer.^4,5^ The EBCTCG meta-analysis demonstrated that breast-conserving surgery combined with external radiotherapy was effective in reducing the local recurrence rate of breast cancer, but approximately 19.3% of patients still experienced recurrence within 10 years.^6^ It has been found that 80% to 90% of local recurrences after breast-conserving treatment occur in the tumour bed or its surrounding areas.^7–9^ At present, it is unadvisable to blindly extend the excision of breast tissue around the tumour to reduce the local recurrence rate, as this would severely damage the appearance of the breast and defeat the key significance of breast conservation. Therefore, it is important to find a new radiotherapy method to enhance local tumour control while ensuring complete excision of the tumour.

Conventional radiotherapy after breast-conserving surgery is administered in the form of conventional segmentation (45–50 Gy at 1.8–2.0 Gy/dose, once per day, five times per week, with or without a tumour bed boost of 10.0–16.0 Gy), but the current conventional form of external irradiation has been challenged by the continuous advances in breast cancer research. Studies have shown that conventional radiotherapy has the disadvantages of poor compliance, long intervals, and inaccurate tumour bed localisation.^10–12^ Therefore, it is necessary to adopt new radiotherapy methods to further improve the local tumour control rate after breast-conserving surgery for early-stage breast cancer.

In recent years, an increasing number of breast cancer centres have introduced intraoperative radiotherapy (IORT) into breast-conserving treatments and have investigated its indications, modalities, doses, efficacy, and prognosis. There are currently two techniques of IORT: electron intraoperative therapy (ELIOT) using a linear electron accelerator, and targeted intraoperative radiotherapy (TARGIT) using the INTRABEAM system with 50 kV low energy x-rays.^13,14^ The idea behind IORT in breast-conserving surgery is to deliver a high, single dose in the most precise way to the areas with the highest risk of subclinical, tumour cell contamination through direct visualisation. The dose of IORT is usually 8–21 Gy, which has the advantages of shortening the treatment course, precisely locating the target area, and improving the radiobiological effect on the tumour, thus compensating for the shortcomings of conventional radiotherapy.

There are two main treatment modalities for IORT; the first is the more radical modality of simply replacing all postoperative, external radiotherapy with a single, high-dose, intraoperative, local irradiation. The second is the more conservative modality of retaining postoperative whole breast irradiation (WBI) and replacing external tumour bed boost with a single, high-dose, intraoperative, local irradiation. Representative studies of a single IORT as a substitute for all postoperative, external radiotherapy include the ELIOT and TARGIT-A trials. More recently, the ELIOT trial published updated results with a median follow-up of 12.4 years, demonstrating a significant 10.2% increase in the 15-year IBTR rate in the single IORT group compared with the external irradiation radiotherapy group.^15,16^ Similarly, preliminary results from TARGIT-A trial showed that the local control rate of IORT alone after breast-conserving surgery was noninferior to that of postoperative external radiotherapy in patients aged ≥45 years with early-stage breast cancer; however, the 5-year local recurrence rate was significantly higher in the IORT group than in the external, radiotherapy group.^17,18^ Therefore, the effectiveness of IORT alone remains controversial.

Although IORT can compensate for the shortcomings of conventional radiotherapy to a certain extent, there are limitations to IORT: for example, small area of irradiation, small total dose, short duration, and insufficient evidence. Therefore, optimising and combining IORT with conventional external radiotherapy has become a major challenge in the treatment of early breast cancer after breast-conserving surgery. To address this challenge, some studies have explored the combination of IORT and conventional external radiation radiotherapy, demonstrating that IORT as a tumour bed boost (IORT boost) combined with postoperative WBI is not inferior to postoperative WBI combined with external radiation tumour bed boost (EBRT boost) in terms of local tumour control rate.^19–28^ This new radiotherapy modality combines the advantages of IORT with conventional radiotherapy, which can reduce the duration of radiotherapy to a certain degree. To date, no corresponding meta-analyses have sufficiently investigated this novel modality. Thus, we conducted this meta-analysis to evaluate the efficacy, cosmetic results, and safety of IORT as a tumour bed boost combined with WBI compared with traditional radiotherapy in women with early-stage breast cancer and breast-conserving surgery.

## Materials and Methods

### Literature Search

We searched in PUBMED, MEDLINE, EMBASE, Web of Science, and Cochrane Library by using the following phrases: “breast cancer”; “intraoperative radiotherapy”; “IORT”; and “boost.” Medical subject headings and free terminology were used for each electronic search. There were no language-based restrictions. The search period was from the establishment of the database until December 31, 2022. References of the included studies also were reviewed to identify potentially eligible articles. Two reviewers (JH and SC) independently conducted literature searches. The detailed searching strategy was listed in the Supplemental Data 1.

## Study Selection

This meta-analysis was performed in accordance with the Preferred Reporting Items for Systematic Review and Meta-analysis (PRISMA) 2020 statement.^29^ Studies that assessed the efficacy, cosmetic outcome, and safety of IORT boost combined with postoperative WBI and compared the findings with those of conventional radiotherapy (with or without tumour bed boost) after breast-conserving surgery in patients with early-stage breast cancer were included. The eligibility criteria are listed below.

### Population

- Women with early-stage breast cancer who underwent breast-conserving treatment, including those with stage T1-2N0-3M0;
- Experimental group: IORT boost combined with postoperative WBI;
- Control group: Conventional external beam radiotherapy (EBRT), including WBI using external irradiation with or without tumour bed boost;
- Outcomes: Local recurrence rates (LRRs), survival outcomes, cosmetic outcomes, and toxicity;
- Study design: Randomised, clinical trials (RCTs) and other comparative studies with control groups.

The following studies were excluded: studies using single IORT as an alternative to whole breast external radiation radiotherapy; studies with duplicate data or full text not available or no available data; single-arm studies; and reviews. For multiple publications of the same study, publications with the most recent data and applicable information were used.

### Data Extraction and Quality Assessment

Two authors (LY and YS) independently performed data extraction and quality assessment. Disagreements were resolved through consensus. Data extracted from each study were as follows: first author, date of publication, study design, study group, study sample size, study intervention, patient characteristics, median follow-up time, and outcomes. Outcomes included local recurrence and distant metastasis rates, disease-free survival (DFS), cosmetic outcomes, and toxicity. The quality of the RCTs was assessed according to the PEDro scale, with a maximum score of 11.^30,31^ The quality of non-RCTs was evaluated according to the Methodological Index of Non-randomised Studies (MINORS), with a maximum score of 24.^32^

### Statistical Analysis

Statistical analyses were performed by using RenMan 5.3 software. The incidence of the outcome indicators analysed in the included studies were all low. Risk ratios (RRs) and 95% confidence intervals (CIs) were used as effect indicators for local recurrence rate, distant metastasis rate, DFS, cosmetic outcome, and toxicity. Statistical heterogeneity between the results of the included studies was analysed by using a *Q*-test (test level set at *α* = 0.10). The results were analysed by using a fixed-effects model if the heterogeneity was small (*I*^2^ < 50%), otherwise a random-effects model or descriptive analysis was used. A Z-test was used to test the significance of the pooled RRs (considered significant at *p* < 0.05). The results of the tests are presented in a forest plot. In addition, we performed sensitivity analyses by excluding studies individually to test the stability of the results of our meta-analysis. Finally, funnel plots were used to determine whether there was publication bias for the main outcome indicators.

## Results

### Study Selection

**Fig. 1:**
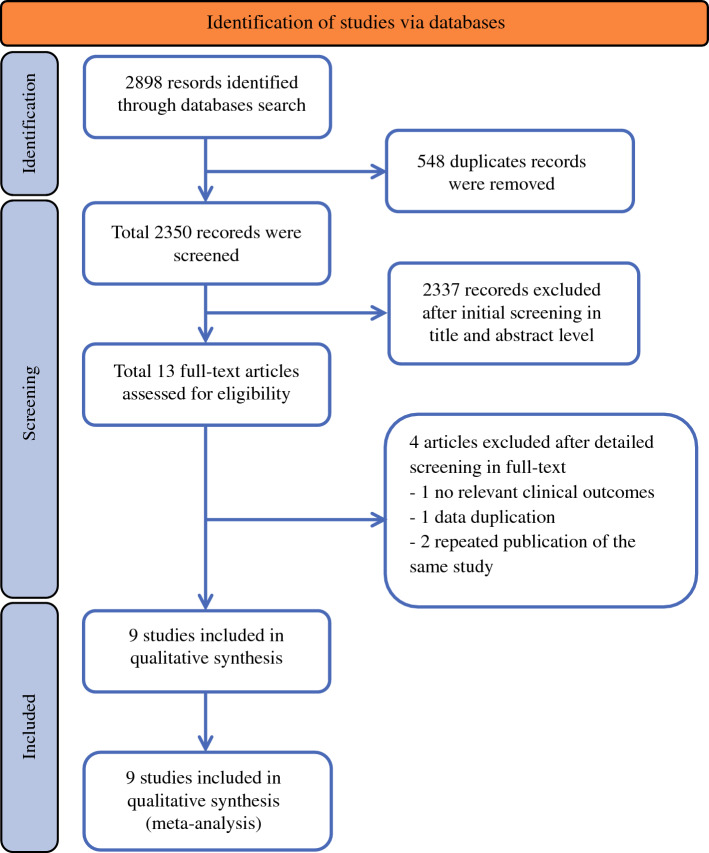
PRISMA flowchart: details of the literature search and selection process

We identified 2898 records. After excluding duplicate records, we retrieved 2350 articles, which were further filtered by screening titles and abstracts. Of these articles, 2337 were excluded for the following reasons: use of intraoperative IORT as an exclusive alternative to total external breast irradiation radiotherapy; lack of a control group in the study; or no available results, reviews, or comments. Finally, through further full-text reading and screening, a total of nine studies met our eligibility criteria and were selected for final analysis.^19–27^ The study selection process is illustrated in Fig. 1.

### Study Characteristics and Quality Assessment

The nine included studies comprised one RCT and eight non-RCTs, with a total of 3219 patients. Of these, 1832 patients were in the IORT group and 1387 patients in the EBRT group. The baseline characteristics of each study were balanced between the two groups. Most of the studies began in 2000 or later and were published between 2006 and 2021. The sample sizes ranged from 46 to 1646 cases. Except for the Fastner and Hashemi studies, the mean or median age of the enrolled patients was 50 years or older. The tumour size was inconsistent in each study but was generally less than 3 cm. The follow-up time also was inconsistent in each study; the shortest follow-up time was only 4–6 months, and the longest median follow-up time was up to 12 years. Overall, the methodological quality of the nine included studies was acceptable. One RCT had a score of 8 on the PEDro scale^19^; the scores were lost because the patients and investigators were not blinded. Six prospective cohort studies had MINORS scores of 19–20^22–27^ whereas the other two, retrospective, case-control studies had MINORS scores of 18.^20,21^ Table 1 presents the quality assessment of the nonrandomized studies included in this meta-analysis. We considered that all studies have a low risk of bias. The main characteristics and quality assessment results of the nine included studies are presented in Table 2.

**Table 1:** Results of the methodological index for nonrandomized studies

Study	Item												Total
	(1)	(2)	(3)	(4)	(5)	(6)	(7)	(8)	(9)	(10)	(11)	(12)	
Reitsamer 2006 [22]	2	2	2	2	0	2	2	0	2	2	2	2	20
Kraus-Tiefenbacher 2006 [25]	2	2	2	2	0	2	2	0	2	2	1	2	19
Welzel 2010 [24]	2	2	2	2	0	2	2	0	2	2	2	2	20
Sperk 2012 [23]	2	2	2	2	0	2	2	0	2	2	1	2	19
Fastner 2014 [20]	2	2	0	2	0	2	2	0	2	2	2	2	18
Kolberg 2017 [21]	2	2	0	2	0	2	2	0	2	2	2	2	18
Sorrentino 2018 [26]	2	2	2	2	0	2	2	0	2	2	2	2	20
Hashemi 2021 [27]	2	2	2	2	0	2	2	0	2	2	2	2	20

0, not reported; 1, reported but inadequate; 2, reported and adequate. Items: (1) clearly stated goal, (2) inclusion of consecutive patients, (3) prospective collection of data, (4) endpoints appropriate to goal of study, (5) unbiased assessment of study endpoint, (6) follow-up period appropriate to goal of study, (7) loss to follow-up less than 5%, (8) prospective calculation of study size, (9) adequate control group, (10) contemporary groups, (11) baseline equivalence of groups, and (12) adequate statistical analyses

**Table 2:** Main characteristics and quality assessment of included studies

Clinical trials	Dates of accrual	Study design	Age (yr)	Tumour size	Nodal stage	Sample size	Arms	Intervention	Median follow-up	Outcome	Quality assessment
Ciabattoni 2021 [19]	1999 –2004	RCT	Median IORT: 56.3 EBRT: 56.2	IORT (n): T1: 96 T2: 28 T3-T4: 1 Missing: 0 EBRT (n): T1: 79 T2: 22 T3-T4: 1 Missing: 8	IORT: N0: 82 N1: 38 N2: 2 N3: 1 Missing: 2 EBRT: N0: 68 N1: 32 N2: 1 N3: 1 Missing: 8	235	IORT (N = 125) EBRT (N = 110)	IORT: Single dose of 10 Gy to the tumour bed using mobile linear accelerator Novac7+whole breast irradiation (50 Gy). EBRT: Whole breast irradiation (50 Gy) with an external boost (10 Gy).	12 yr (range 10–16 yr)	Local recurrence; Distant recurrence; Cosmetic outcome; Toxicity; Disease-free survival; Overall survival	8^a^
Fastner 2014 [20]	2002 –2007	Non-RCT	Median IORT: 48 EBRT: 53.5	Median (cm) IORT: 3.40 EBRT: 2.60	IORT: N0: 47 N1: 29 N2: 5 N3: 0 EBRT: N0: 12 N1: 7 N2: 4 N3: 3	107	IORT (N = 81) EBRT (N = 26)	IORT: Single dose of 9 Gy (10 Gy [Dmax]) to the tumour bed using electrons+whole breast irradiation (51–57 Gy). EBRT: Whole breast irradiation (51–57 Gy) with an external boost (6–16 Gy).	IORT: 59 mo (range, 3–115) EBRT: 67.5 mo (range, 13–120)	Local control; Locoregional control; Metastasis-free survival; Disease-specific survival; Overall survival	18^b^
Sperk 2012 [23]	2002 –2008	Non-RCT	Median IORT: 62 EBRT: 69	IORT (n): yT0: 1 T1: 128 T2: 66 Unknown: 1 EBRT (n): T1: 47 T2: 8	IORT: N0: 144 N1: 36 N2: 14 Unknown: 2 EBRT: N0: 44 N1: 10 N2: 1	251	IORT (N = 196) EBRT (N = 55)	IORT: Single dose of 20 Gy to the tumour bed using 50 kV x-rays+whole breast irradiation (46–50 Gy). EBRT: Whole breast irradiation (56 Gy/28 fx).	IORT: 52 mo EBRT: 42 mo	Toxicity; Local recurrence; Overall survival	19^b^
Welzel 2010 [24]	2002 –2006	Non-RCT	Median IORT: 67 EBRT: 66	Tumor size <2 cm, n (%) IORT: 14 (61%) EBRT: 17 (74%)	IORT: N0: 20 N1: 3 EBRT: N0: 23 N1: 0	46	IORT (N = 23) EBRT (N = 23)	IORT: Single dose of 20Gy to the tumour bed using 50 kV x-rays+whole breast irradiation (46/50 Gy). EBRT: Whole breast irradiation (56 Gy/28 fx).	IORT: 47 mo EBRT: 44 mo	Quality of life; Toxicity; Disease-free survival	20^b^
Reitsamer 2006 [22]	1996 –2001	Non-RCT	Median IORT: 59.03 EBRT: 57.07	IORT (n): T1a: 3 T1b: 15 T1c: 112 T2: 60 EBRT (n): T1a: 6 T1b: 20 T1c: 102 T2: 60	IORT: N (-): 117 N1mic: 14 N (+): 59 EBRT: N (-): 126 N1mic: 8 N (+): 54	378	IORT (N = 190) EBRT (N = 188)	IORT: 9 Gy prescribed to the 90% isodose using 4–18 MeV electrons+whole breast irradiation (51/56.1 Gy). EBRT: Whole breast irradiation (51/56 Gy) with an external boost (12 Gy).	IORT: 51.1 mo EBRT: 81 mo	Ipsilateral breast tumour recurrence; Distant recurrence; Disease-free survival	20^b^
Kraus- Tiefenbacher 2006 [25]	2002 –2005	Non-RCT	Median IORT: 63 EBRT: 56	Median (cm) IORT: 1.50 EBRT: 1.70	IORT: N0: 61 N1: 18 N2: 3 N3: 2 EBRT: N0: 29 N1: 17 N2: 4 N3: 0	137	IORT (N = 84) EBRT (N = 53)	IORT: Single dose of 20 Gy to the tumour bed using 50-kV x-rays+whole breast irradiation (46/23 fx). EBRT: Whole breast irradiation (three patients with ductal carcinoma *in situ* were treated with a total dose of 50 Gy, and 34 patients were treated with 56 Gy. Additionally, 16 patients younger than age 50 yr were treated with 50 Gy WBRT followed by a 14–16 Gy electron boost) (50–66 Gy).	4–6 mo	Cosmetic outcome; Toxicity	19^b^
Kolberg 2017 [21]	2010–2011	Non-RCT	Mean IORT: 54.9 EBRT: 57.8	Mean (cm) IORT: 2.16 EBRT: 2.59	IORT: N (-): 28 N (+): 33 EBRT: N (-): 25 N (+): 30	116	IORT (N = 61) EBRT (N = 55)	IORT: Single dose of 20 Gy to the tumour bed using 50-kV x-rays+whole breast irradiation (50 Gy/25 Fx). EBRT: Whole breast irradiation (50 Gy/25 Fx) with an external boost (10 Gy/16 Gy).	49 mo	Local recurrence; Local recurrence-free survival; Disease-free survival; Distant disease-free survival; Breast cancer mortality; Non-breast cancer mortality; Overall mortality	18^b^
Hashemi 2021 [27]	2013–2019	Non-RCT	Median IORT: 40-50 EBRT: 40-50	Median (cm) IORT: ≤2.5 cm EBRT: ≤2.5 cm	Negative	1646	IORT (N = 989) EBRT (N = 657)	IORT: ①12 Gy as a boost dose by making flaps in breast tissue around the tumour cavity with a maximum thickness of 2 cm, using light intraoperative accelerator (LIAC), a mobile linear accelerator delivering energy levels of 6–12 MeV electrons+whole breast irradiation (45-50 Gy). ②20 Gy intraoperative x-ray radiation therapy of 50 kV was delivered to breast tissue around the tumour cavity+whole breast irradiation (45-50 Gy) EBRT: Whole breast irradiation (45-50 Gy) with an external boost (10 Gy).	IORT: 34.5 mo EBRT: 40.18 mo	Local recurrence; Distant recurrence; Disease-free survival; Overall survival	20^b^
Sorrentino 2018 [26]	2011–2016	Non-RCT	Mean IORT: 61.4 (±11.2) EBRT: 65.5 (±8.9)	IORT (n): Tx: 0 Tis: 2 T1a: 3 T1b: 20 T1c: 51 T2: 7 EBRT (n): Tx: 1 Tis: 9 T1a: 24 T1b: 62 T1c: 99 T2: 25	IORT: N (-): 73 N (+): 10 EBRT: N (-): 185 N (+): 35	303	IORT (N = 83) EBRT (N = 220)	IORT: 12 Gy as a boost using a linear accelerator (LIAC, SIT®)+hypofractionated scheme of 13 daily fractions of 2.85 Gy, up to a total dose of at 37.05 Gy. EBRT: 45 Gy in 20 fractions plus a concomitant 5 Gy boost in four weekly fractions (1.25 Gy per fraction), usually delivered with electrons.	IORT: Mean 37.1 (±13.8) mo EBRT: Mean 33.5 (±17.4) mo	Local recurrence; Distant recurrences; Adverse effects and toxicities; Evaluation of quality of life and self-perception of body image; Changes in working and normal daily activities	20^b^

^a^According to the PEDro scale

^b^According to the Methodological Index for Nonrandomised Studies (MINORS)

## Meta-analysis Results and Sensitivity Analysis

### Local Recurrence Rate

**Fig. 2:**
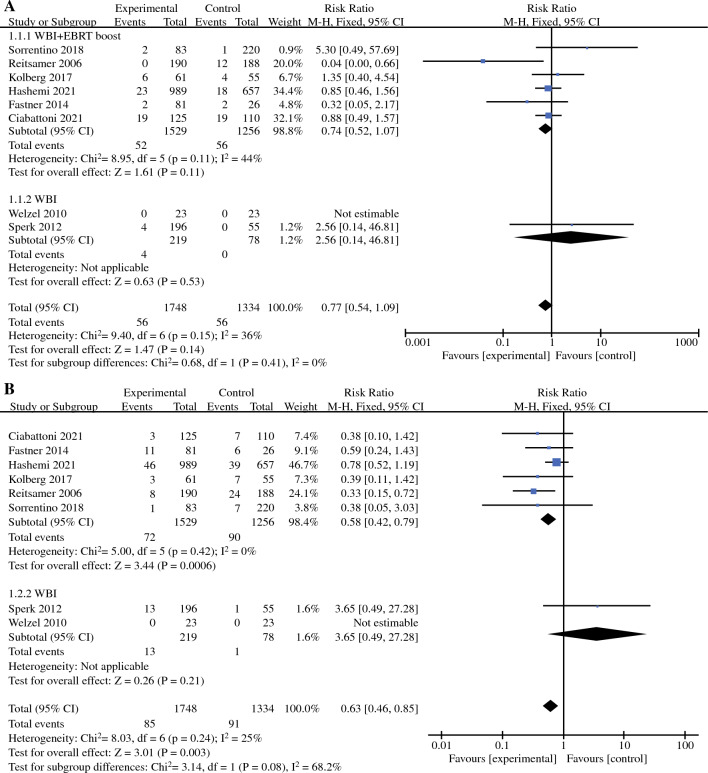

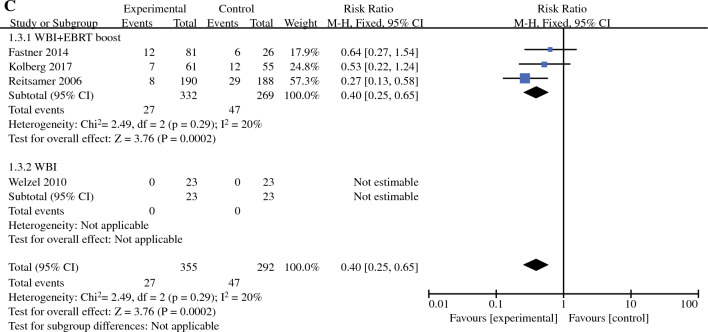
**a** Forest plot of local recurrence rate; **b** Forest plot of distant metastases rate; **c** Forest plot of disease-free survival

Local recurrence included ipsilateral intramammary, cutaneous, and chest wall recurrence. Eight of the nine included studies reported RRs for local recurrence rates (LRR) data (one RCT and seven non-RCTs, with a total of 3082 patients).^19–24,26,27^ Because of the small heterogeneity (*I*^2^ = 36%, *P* = 0.15), a fixed-effects model was used for meta-analysis. The pooled RR for LRR showed no statistically significant difference between the IORT boost+WBI group and the conventional radiotherapy group (RR = 0.77, 95% CI: 0.54–1.09, *P* = 0.14), but LRR in the IORT boost+WBI group showed a trend towards improvement. In subgroup analysis, compared with the EBRT boost+WBI group, LRR in the IORT boost+WBI group also tended to improve, but there was still no statistically significant difference between the two groups (RR = 0.74, 95% CI: 0.52–1.07, *P* = 0.11; Fig. 2a). We performed sensitivity analyses by excluding these studies individually and found that the heterogeneity in LRR was mainly derived from the article by Reitsamer (2006). When we excluded this article, no significant heterogeneity was found between the remaining seven studies (*I*^2^ = 0%, *P* = 0.52), and there was no statistically significant change in the effect values for recombination (RR = 0.95, 95% CI: 0.65–1.38, *P* = 0.77).

### Distant Metastases Rate

Similarly, all eight studies that provided LRR data also provided RRs for distant metastasis rate (DMR) with low heterogeneity (*I*^2^ = 25%, *P* = 0.24), so the same fixed-effects model was used for meta-analysis.^19–24,26,27^ The pooled RR showed that DMR was significantly lower in the IORT boost+WBI group than in the conventional radiotherapy group (RR = 0.63, 95% CI: 0.46–0.85, *P* = 0.003). Furthermore, a subgroup analysis showed that the DMR in the IORT boost+WBI group also was significantly lower than the DMR in the EBRT boost+WBI group (RR = 0.58, 95% CI: 0.42–0.79, *P* = 0.0006; Fig. 2b). Sensitivity analysis revealed that the heterogeneity in DMR mainly originated from the article by Sperk (2012). When we excluded this article, no significant heterogeneity was found between the remaining seven studies (*I*^2^ = 0%, *P* = 0.42), and no statistically significant change in effect values was observed for the recombination (RR = 0.58, 95% CI: 0.42–0.79, *P* = 0.0006).

### Disease-Free Survival

Disease-free survival (DFS) was defined as the proportion of patients who did not experience any recurrence. Six studies provided data on DFS, but only four non-RCTs provided RRs for DFS data (647 patients in total).^20–22,24^ The other two studies provided 5- or 10-year cumulative survival rates only.^19,27^ The heterogeneity of DFS was small (*I*^2^ = 20%, *P* = 0.29); therefore, the fixed effects model was used for meta-analysis. The pooled RR showed that DFS was significantly better in the IORT boost+WBI group than in the conventional radiotherapy group (RR = 0.40, 95% CI: 0.25–0.65, *P* = 0.0002). In a subgroup analysis, it was concluded that DFS also was significantly better in the IORT boost+WBI group than in the EBRT boost+WBI group (RR = 0.40, 95% CI: 0.25–0.65, *P* = 0.0002; Fig. 2c). Sensitivity analysis showed that the heterogeneity in DFS mainly originated from the article by Reitsamer (2006). When we excluded this article, no significant heterogeneity was found between the remaining seven studies (*I*^2^ = 0%, *P* = 0.75). There was, however, a statistically significant change in the effect values for the recombination (RR = 0.57, 95% CI: 0.31–1.06, *P* = 0.08). However, considering that the article by Reitsamer (2006) fully met the inclusion criteria, the quality of the article was well assessed, and the overall heterogeneity was low, we still included this article in the pooled analysis of DFS.

### Cosmetic Outcome

**Fig. 3:**
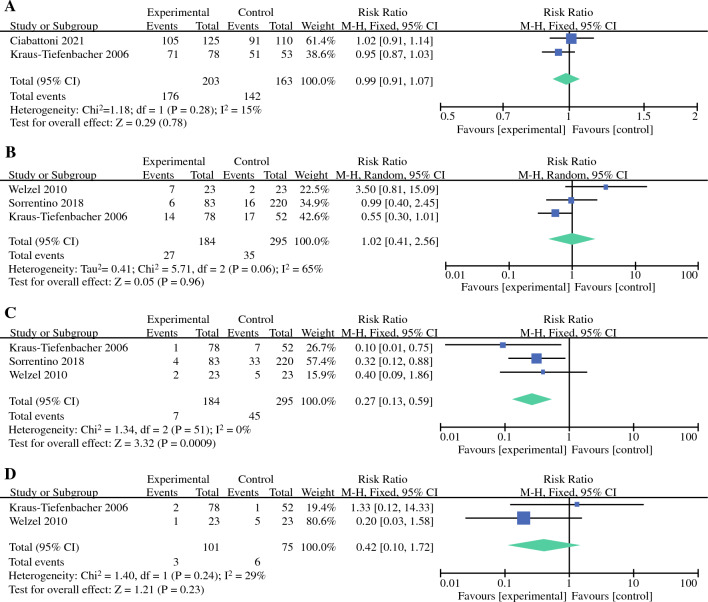
Forest plots of cosmetic outcome and toxicity: **a** cosmetic outcome; **b** fibrosis; **c** oedema; **d** hyperpigmentation

Two studies provided RRs for cosmetic outcomes (one RCT and one non-RCT; 366 patients in total).^19,25^ The heterogeneity of cosmetic outcomes was small (I^2^ = 15*%, P* = 0.28); therefore, the fixed-effects model was used for meta-analysis. The pooled RR for cosmetic outcome showed no significant difference between the IORT boost+WBI group and the conventional radiotherapy group (RR = 0.99, 95% CI: 0.91–1.07, *P* = 0.78; Fig. 3a).

### Fibrosis

Three studies provided RRs for fibrosis (479 patients in total).^24–26^ The heterogeneity was greater for fibrosis (*I*^2^ = 65%, *P* = 0.06); therefore, a random-effects model was used for meta-analysis. The pooled RR showed no significant difference between the IORT boost+WBI group and the conventional radiotherapy group (RR = 1.02, 95% CI: 0.41–2.56, *P* = 0.96; Fig. 3b). Sensitivity analysis revealed that the heterogeneity of fibrosis mainly originated from the article by Welzel (2010). When we excluded this article, we found that the heterogeneity could be significantly reduced (*I*^2^ = 13%, *P* = 0.28) and that there was no statistically significant change in the effect values for the recombination (RR = 0.67, 95% CI: 0.39–1.17, *P* = 0.16).

### Oedema

All three studies that provided fibrosis data also provided RRs for oedema with no significant heterogeneity (*I*^2^ = 0%, *P* = 0.51).^24–26^ Thus, a fixed-effects model was used for meta-analysis. The pooled RR indicated that the incidence of oedema was significantly lower in the IORT boost+WBI group than in the conventional radiotherapy group (RR = 0.27, 95% CI: 0.13–0.59, *P* = 0.0009; Fig. 3c).

### Hyperpigmentation

Two studies provided RRs for hyperpigmentation (176 patients in total).^24,25^ The heterogeneity of hyperpigmentation was small (*I*^2^ = 29%, *P* = 0.24); therefore, a fixed-effects model was used for meta-analysis. The pooled RR showed no significant difference between the IORT boost+WBI group and the conventional radiotherapy group (RR = 0.42, 95% CI: 0.10-1.72, *P* = 0.23; Fig. 3d).

### Subgroup Analysis

**Fig. 4:**
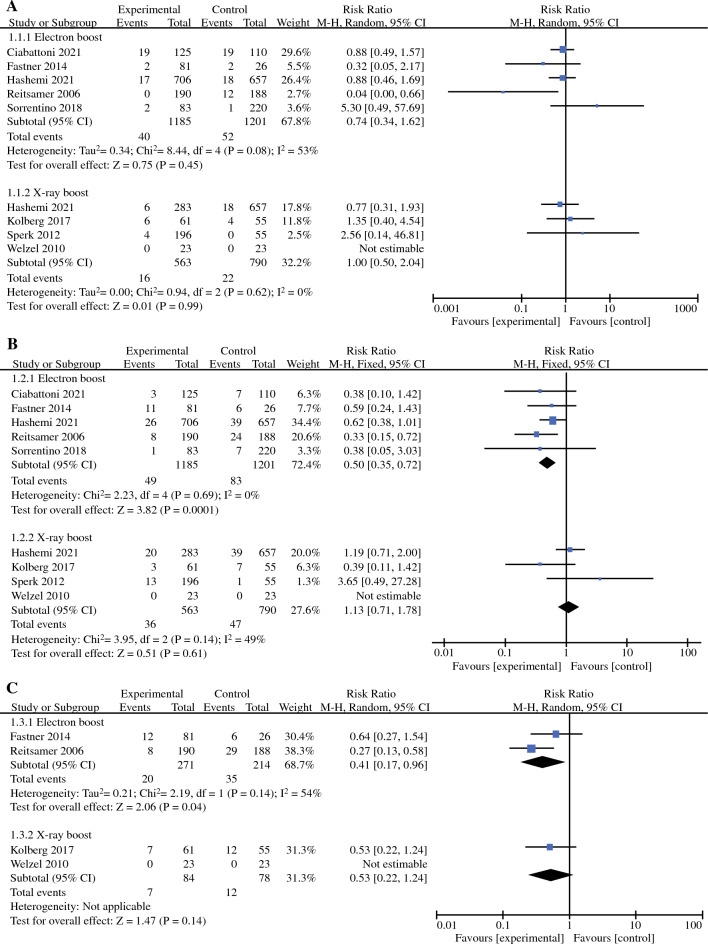
Forest plots of LRR, DMR, and DFS for each of the two different IORT boost+WBI modalities versus conventional radiotherapy: **a** LRR; **b** DMR; (**c**) DFS

We performed a subgroup analysis to explore whether the LRR, DMR, and DFS differed between electron boost+WBI and x-ray boost+WBI groups compared with the corresponding outcomes of conventional radiotherapy. The pooled RRs for LRR showed no statistically significant difference between the two different boosts + WBI compared with conventional radiotherapy respectively (RR = 0.74, 95% CI: 0.34–1.62, *P = *0.45; RR = 1.00, 95% CI: 0.50–2.04, *P = *0.99), but LRR in the electron boost+WBI group showed a trend towards improvement. Electron boost+WBI group had significantly better DMR and DFS than did the conventional radiotherapy group (RR = 0.50, 95% CI: 0.35–0.72, *P = *0.0001; RR = 0.41, 95% CI: 0.17–0.96, *P = *0.04). However, the x-ray boost+WBI group did not effectively improve the DMR and DFS (RR = 1.13, 95% CI: 0.71–1.78, *P = *0.61; RR = 0.53, 95% CI: 0.22–1.24, *P = *0.14; Fig. 4a, b, c).

### Assessment of Publication Bias

**Fig. 5:**
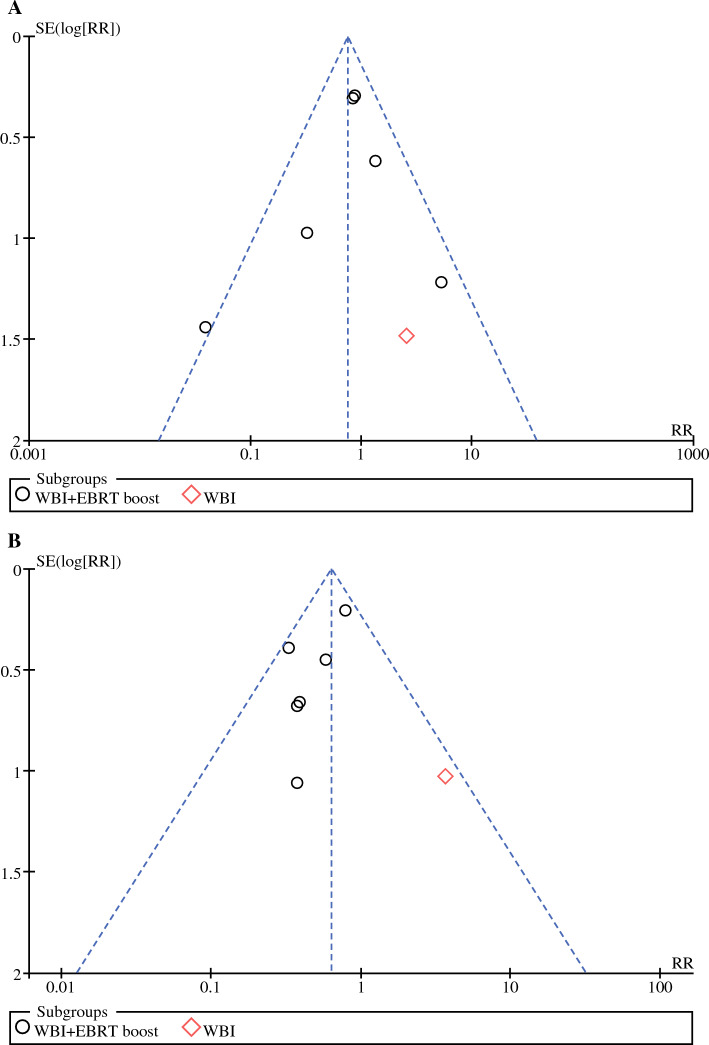
Funnel plots of publication bias for LRRs and DMRs in the IORT boost+WBI group versus conventional radiotherapy group: **a** LRRs; **b** DMRs

Our study assessed publication bias in eight articles for local recurrence and distant metastasis rates due to the variable number of included studies for each outcome indicator.^19–24,26,27^ Funnel plots for the two evaluation indicators both showed a more symmetrical left-right pattern, suggesting a low likelihood of publication bias (Fig. 5a, b). Few articles were included for all other outcome indicators; therefore, publication bias was not assessed.

## Discussion

Many single-arm studies have demonstrated the efficacy and safety of IORT boost+WBI;^33–43^ however, comparative studies on IORT boost+WBI and conventional radiotherapy are still lacking. Through a search of major databases, we screened nine comparative studies of IORT boost+WBI versus conventional radiotherapy, with varying results. In this meta-analysis of nine studies with a total of 3219 patients, the pooled results showed that among women with early-stage breast cancer who underwent breast-conserving surgery, those who received IORT boost+WBI had significantly better DMR and DFS than those who received conventional radiotherapy (with or without the tumour bed boost) (RR = 0.63, 95% CI: 0.46–0.85, *P = *0.003; RR = 0.40, 95% CI: 0.25–0.65, *P = *0.0002). Although LRR was not significantly different between the two groups, the IORT boost+WBI group showed a trend towards benefit (RR = 0.77, 95% CI: 0.54–1.09, *P = *0.14).

In this meta-analysis, we did not find significantly better LRR in the IORT boost+WBI group than in the conventional radiotherapy group, which may be related to the generally short follow-up period of the included studies (3–5 years in most studies). Moreover, all the studies had different inclusion criteria and included different proportions of various subtypes of breast cancer, leading to a bias in the risk of recurrence, and the studies were conducted in different contexts. This indicates some variations in systemic treatment, which may affect the final outcome. However, it is undeniable that the patients included in these studies were generally at low risk of recurrence and had small tumour sizes; therefore, the LRRs in these patients were low even with conventional radiotherapy, making it difficult to achieve a statistically significant advantage in the experimental group and highlighting the need for larger sample sizes and a longer follow-up period.

As shown in Table 2, our meta-analysis included patients with low- to high-risk, early-stage breast cancer staged as T1-2N0-3M0. From Fig. 2a, we observed that IORT boost+WBI tended to be have a more beneficial effect on LRR than did conventional radiotherapy. In addition, a large multicentre, prospective, randomised, clinical study for breast cancer patients with a high risk of recurrence is currently ongoing: the TARGIT-B trial. The inclusion criteria for the TARGIT-B trial are poor characteristics, such as age younger than 46 years, invasive lobular carcinoma, neoadjuvant chemotherapy, and age older than 45 years with grade 3 or ER/PR-negative or lymph node positive. Its primary outcome is local tumour control, with secondary outcomes, including the site of breast recurrence, survival, toxicities, and quality of life. We look forward to the final results of the study. Nonetheless, there is still a lack of relevant, prospective, randomised, clinical studies for those low-risk patients who are not eligible for the TARGIT-B trial.

In our meta-analysis, we were surprised to observe that both DMR and DFS were significantly better in the IORT boost+WBI group than in the conventional radiotherapy group. This result is perhaps more meaningful than that of LRR. This is because it is well known that a large proportion of deaths in breast cancer patients are due to distant metastases, leading to dysfunction of the involved vital organs and, ultimately, death. Only a small proportion of nonbreast–cancer-specific deaths are due to the exacerbation of the patient’s own underlying disease. Therefore, the benefit of DMR and DFS in the IORT boost+WBI group gives us hope for prolonged overall survival (OS) or breast cancer-specific OS. As known, conventional radiotherapy is used to inhibit the growth and reproduction of tumour cells by directly damaging their DNA structure through radiation irradiation. However, IORT differs from conventional radiotherapy in terms of the operating method and treatment dose. After a single high dose of irradiation to local tissues, the DNA structure of tumour cells is destroyed, but the surrounding ductal epithelial cells, adipocytes, or vascular endothelial cells also may be damaged to varying degrees. The inflammatory response of local tissues, fibrosis, and impaired microcirculation are factors that affect distant tumour metastasis and are perhaps the most important mechanisms of IORT. However, no study has specifically investigated the mechanisms of IORT, probably because current clinical trials of IORT have not shown satisfactory results. We hope to see the benefits of IORT in terms of OS for patients in future, high-quality, clinical studies.

Notably, four of the five studies that provided OS data showed no benefit in OS or breast cancer-specific OS in the IORT boost+WBI group. The 3-year OS in the IORT boost+WBI group in the Sperk 2012 was 93% compared with 100% in the conventional radiotherapy group. After a median follow-up of 5 years in the Fastner (2014) study, the 6-year OS was 86.4% in the IORT boost+WBI group and 92% in the WBI+EBRT boost group, with no statistical difference between the two groups. In Ciabattoni (2021), after a median follow-up of 12 years, the 5-year and 10-year OS in the IORT boost+WBI group were 94.5% and 91.6%, respectively, compared with 99% and 94.3% in the WBI+EBRT boost group, with no statistical difference between the two groups (*P = *0.377). Hashemi (2021) yielded a 5-year OS of 95.1%, 97.5%, and 97.2% for the EBRT, electron boost, and x-ray boost groups, respectively. In terms of OS, no statistically significant differences were found among the three groups. However, a study by Kolberg (2017) concluded that the IORT boost+WBI group showed a significantly better 5-year OS (96.7% vs. 81.7%, HR = 0.19, 95% CI: 0.04-0.87, *P = *0.016) and nonbreast–cancer-specific OS (100% vs. 89.9%, *P = *0.015) than the conventional radiotherapy group did but showed no significant improvement in breast–cancer-specific OS (96.7% vs. 90.9%, HR = 0.42, 95% CI: 0.08-2.30, *P = *0.30). It is well known that the two peaks of recurrence in breast cancer are 1–3 years after surgery and 7–8 years after surgery. For patients with a lower risk of recurrence, we need a longer follow-up period to observe recurrence. For these studies, a short follow-up period is probably the most important reason for the reported effects on LRR, OS, and breast–cancer-specific OS. Therefore, these studies need a longer follow-up period to further observe the differences in recurrence rates and survival between the two groups.

As mentioned previously, IORT consists of two main systems: a portable, electron-accelerator ELIOT system, and a photon-therapy INTRABEAM system. In our meta-analysis, the ELIOT system alone was used in four studies, the INTRABEAM system alone was used in four studies, and both systems were used in one study. Surprisingly, Hashemi (2021) concluded that 5-year DFS was 89.5%, 92.3%, and 91.3% for the x-ray boost, electron boost, and EBRT groups, respectively.^27^ There was no significant difference in 5-year DFS between EBRT and x-ray boost groups (*P = *0.36) and between EBRT and electron boost groups (*P = *0.26). Nevertheless, there was a significant difference between the x-ray boost and electron boost groups (*P = *0.037), with a better DFS in the electron boost group. Therefore, we performed a subgroup analysis to explore whether the LRR, DMR, and DFS differed between electron boost+WBI and x-ray boost+WBI groups compared with the corresponding outcomes of conventional radiotherapy. As a result, we found that the electron boost+WBI group had significantly better DMR and DFS than did the conventional radiotherapy group, and that there was a trend towards a benefit in LRR in the electron boost+WBI group. However, the x-ray boost+WBI group did not effectively improve the LRR, DMR, and DFS. Undoubtedly, these results are premature, and more multicentre, prospective, randomised, clinical studies are needed to explore the differences in the efficacy and safety of electron boost compared to x-ray boost, as well as basic research to explore the mechanistic differences between the two.

In terms of appearance and toxicity, our meta-analysis yielded no significant differences in cosmetic outcome, fibrosis, and hyperpigmentation between the two groups (RR = 0.99, 95% CI: 0.91–1.07, *P* = 0.78; RR = 1.02, 95% CI: 0.41–2.56, *P* = 0.96; RR = 0.42, 95% CI: 0.10–1.72, *P* = 0.23), but the incidence of oedema was significantly reduced in the IORT boost+WBI group (RR = 0.27, 95% CI: 0.13-0.59, *P = *0.0009). However, the results should be interpreted with caution due to the small number of studies providing cosmetic outcomes and toxicity as well as mild-to-moderate heterogeneity. Furthermore, several previous studies have explored the toxicity and cosmetic outcomes of IORT boost+WBI. The studies concluded that IORT boost+WBI proved to be safe and feasible compared with conventional treatment, showing no treatment-related mortality, no delayed wound healing, or increased infection rates.^19,45–48^ According to research by experts, such as Lemanski^47^ and Salzburg,^49^ most patients were satisfied with the overall cosmetic effect after IORT+WBI treatment. In addition, for patients receiving IORT boost combined with hypofractionated whole breast irradiation (HWBI), the large, prospective, multicentre HIOB trial suggested that the patient’s early tolerance and cosmetic effect were excellent, but we still need to follow the data on its long-term follow-up.^50^

### Advantages and Limitations

The efficacy of IORT alone has been highly questioned, resulting in the extremely slow development of intraoperative radiotherapy. However, IORT as a tumour bed boost combined with WBI is a new radiotherapy modality, and few comparative studies have investigated its efficacy and safety. The strength of our meta-analysis is that it is the first to bring together a systematic review and pooled analysis of relevant clinical studies of IORT boost+WBI compared with conventional radiotherapy. This meta-analysis provides important evidence for the use of IORT as a tumour bed boost. However, our study had some limitations. First, the major limitation of this meta-analysis was that quality scores, patient populations, treatment protocols, and follow-up periods varied across the studies. Second, our meta-analysis included only nine studies involving 3,219 patients. None of these nine studies were blinded, and eight of them were non-RCTs with lower evidence ratings than RCTs, suggesting that our studies are prone to systematic errors, such as selection bias. Moreover, one in nine studies accounted for nearly half of the total number of patients. However, most of the studies were prospective, controlled trials with high-quality scores. Third, as an effect measure of survival data, the statistics of local recurrence, distant recurrence, and especially OS rate were best combined by applying the hazard ratio (HR), 95% CI. However, in the literature included in this study, the majority of the original literature could not extract the time to death and cutoff data. Thus, we used RR instead of HR, which lost some important information compared with HR, meaning that the time factor was not considered. The author suggests that future, clinical trials should improve the follow-up and statistical results to provide more detailed data for the clinical application of IORT. These findings suggest that more high-quality, large-sample, prospective studies are needed to enhance the statistical validity of the combined effect values and validate the reliability of the findings.

## Conclusions

This meta-analyses shows that patients with early-stage breast cancer who receive IORT boost+WBI after breast-conserving surgery show significantly better DMR and DFS than those who receive conventional radiotherapy. IORT boost+WBI also shows a tendency to beneficially effect LRR; electron boost exhibit more pronounced efficacy than x-ray boost. Additionally, the cosmetic outcome and adverse effects of IORT boost+WBI are comparable to conventional radiotherapy.

### Supplementary Information

Below is the link to the electronic supplementary material.

Supplementary file1 (DOCX 1156 kb)
