# Supplementary material for: Intraoperative Radiotherapy as a Tumour-Bed Boost Combined with Whole Breast Irradiation Versus Conventional Radiotherapy in Patients with Early-Stage Breast Cancer: A Systematic Review and Meta-analysis
Source: Ann Surg Oncol. 2023 Jul 28;30(13):8436–52. doi: 10.1245/s10434-023-13955-w (PMC10625949; doi:10.1245/s10434-023-13955-w)
Supplement: Supplementary file 1 — Supplementary file1 (DOCX 1156 kb) [file 10434_2023_13955_MOESM1_ESM.docx]

**Appendix. Supplementary data**

**Supplementary data 1: Systematic Review Searching Record**

**Literature search details**

**Date Restriction: Search literature up to 31 December 2022.**

**Language Restriction: none**

| **Database name** | **No. of records found** | **Date of search** |
| --- | --- | --- |
| **Pubmed** | **1198** | **1 January 2023** |
| **Web of Science** | **632** | **1 January 2023** |
| **Embase** | **644** | **1 January 2023** |
| **Medline**  **Medline in process** | **337** | **1 January 2023** |
| **Cochrane Library** | **87** | **1 January 2023** |

**Total Records Found: 2898**

**Total Records after de-duplication: 2350**

**(548 duplicate records found by Endnote)**

**Pubmed**

**
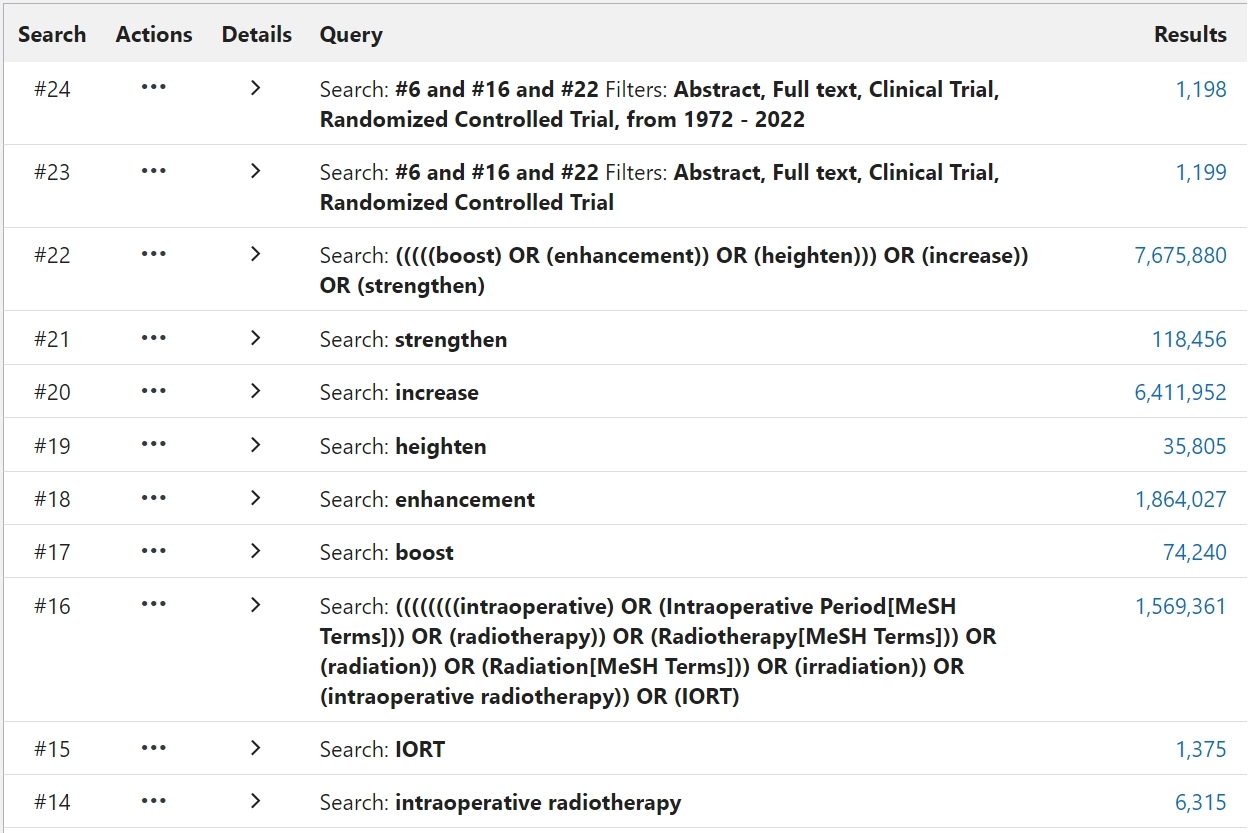

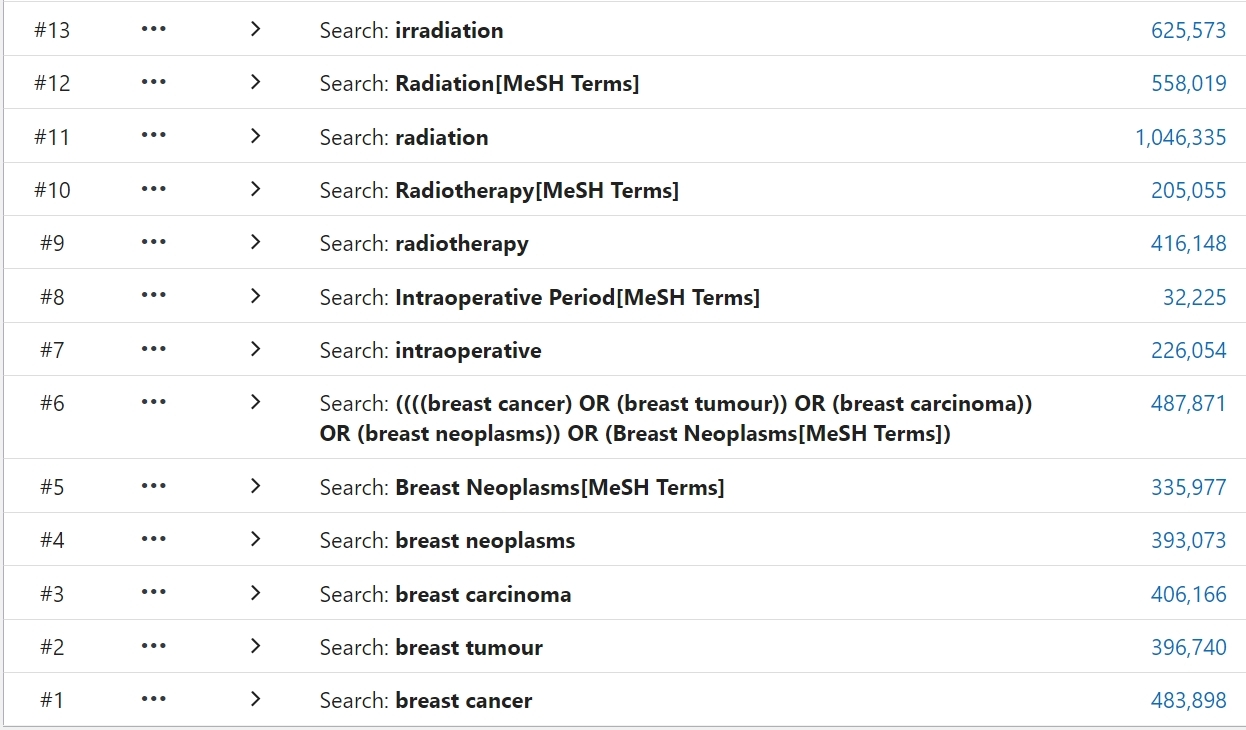
**

**Web of Science**

**
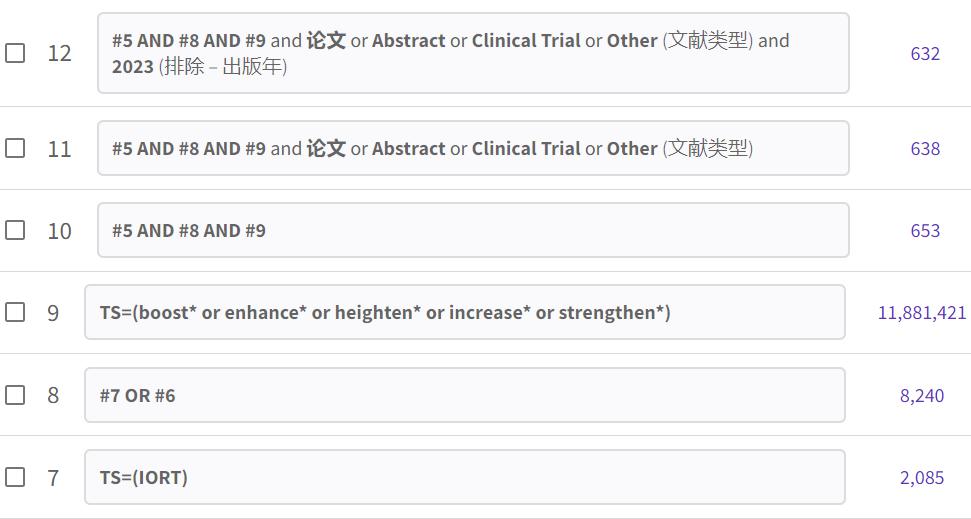

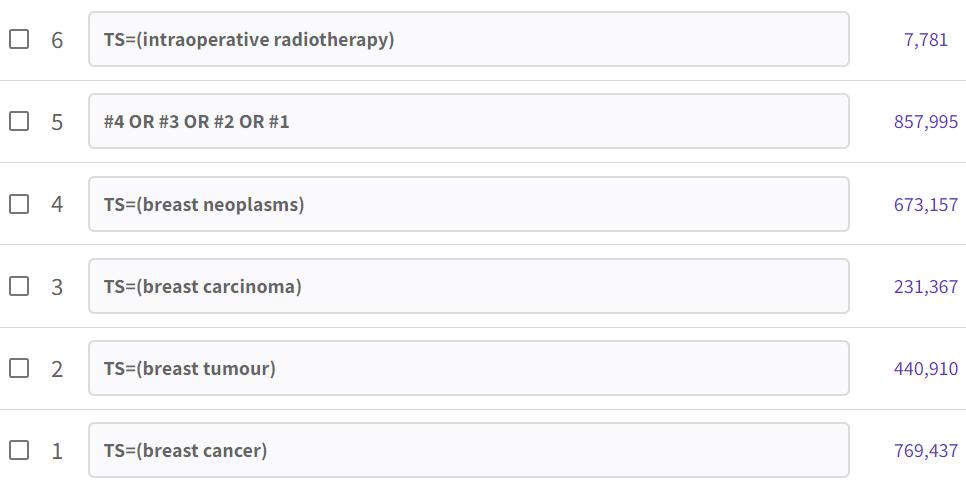
**

**Embase**

**
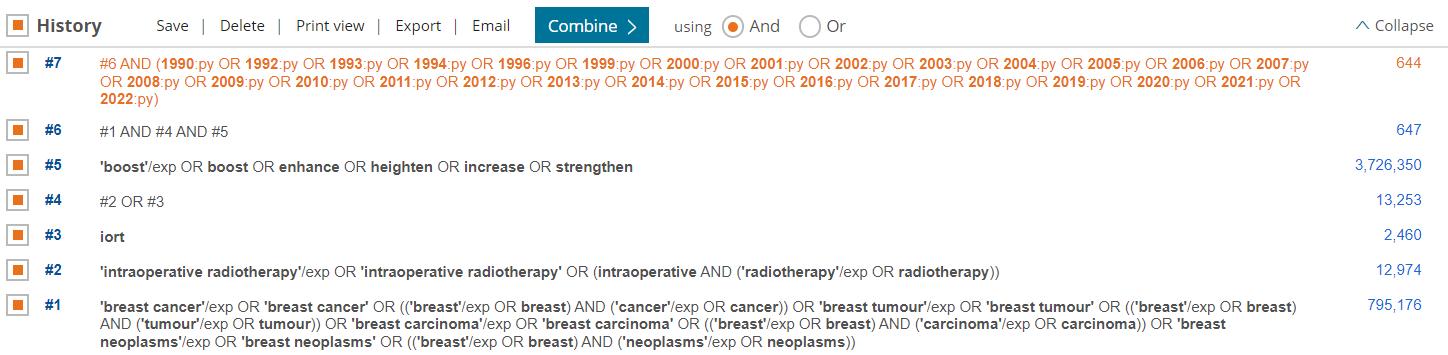
**

**Medline**

**
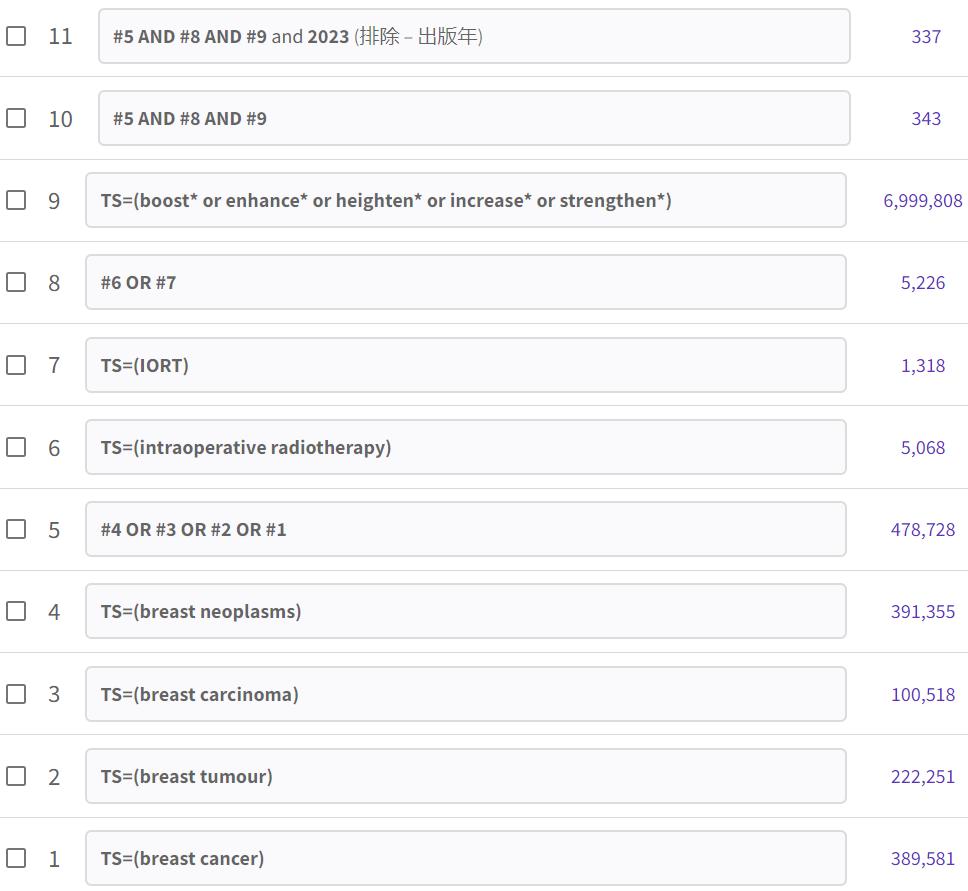
**

**Cochrane Library**

**
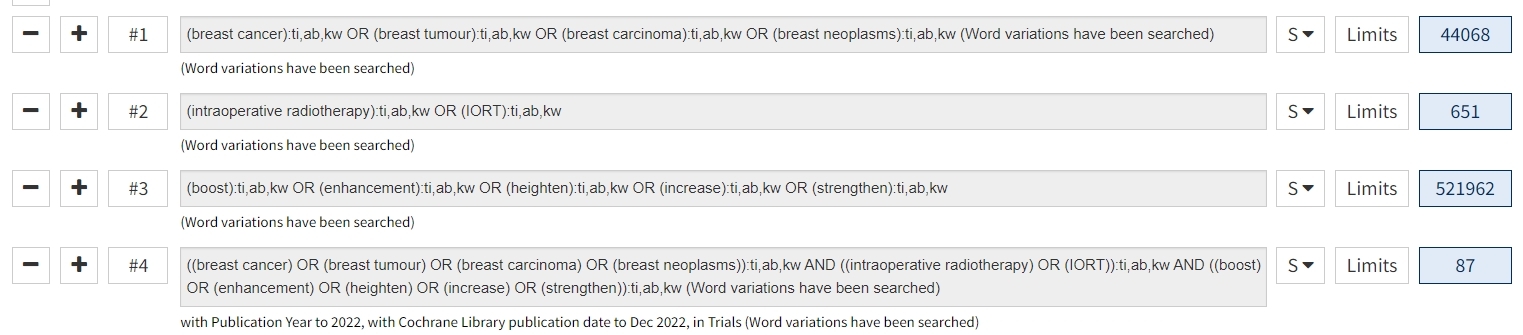
**
